# Importance of strategic management in the implementation of private medicine retailer programmes: case studies from three districts in Kenya

**DOI:** 10.1186/1472-6963-10-S1-S7

**Published:** 2010-07-02

**Authors:** Timothy Abuya, Abdinasir Amin, Sassy Molyneux, Willis Akhwale, Vicki Marsh, Lucy Gilson

**Affiliations:** 1 Kenya Medical Research Institute/Wellcome Trust Centre for Geographic Medicine Research-Coast, Kilifi, Kenya; 2 Malaria Control and Child Survival Department; Population Services International, P.O Box 22591-004000, Nairobi, Kenya; 3 Division of Vector-Borne Diseases, Ministry of Health, Nairobi, Kenya; 4 Centre for Clinical Vaccinology and Tropical Medicine (CCVTM), University of Oxford, Headington, Oxford, OX3 9DU, UK; 5 Health Economics Unit, School of Public Health & Family Medicine, Faculty of Health Sciences University of Cape Town, South Africa

## Abstract

**Abstract:**

**Background**

The home-management of malaria strategy seeks to improve prompt and effective anti-malarial drug use through the informal sector, with a potential channel being the Private Medicine Retailers (PMRs). Previous evaluations of PMR programmes focused on their impact on retailer knowledge and practices, with limited evidence about the influence of implementation processes on the impacts at scale. This paper examines how the implementation processes of three PMR programmes in Kenya, each scaled up within a district, contributed to the outcomes observed. These were a Ministry of Health programme in Kwale district; and two programmes supported by non-governmental organizations in collaboration with government in Kisii Central and Bungoma districts.

**Methods**

The research methods included 24 focus group discussions with clients and PMRs, 19 in-depth interviews with implementing actors, document review and a diary of events. The data were analysed using the combination of a broad policy analysis framework and more specific scaling up/diffusion of innovations frameworks.

**Results**

The Kisii programme, a case study of successful implementation, was underpinned by good relationships between district health managers and a “resource team”, supported by a memorandum of understanding which enabled successful implementation. It had flexible budgetary and decision making processes which were responsive to local contexts, and took account of local socio-economic activities. In contrast, the Kwale programme, which had implementation challenges, was characterised by a complex funding process, with lengthy timelines, that was tied to the government financial management system which constrained implementation Although there was a flexible funding system in Bungoma, a perceived lack of transparency in fund management, inadequate management of inter-organisational relationships, and inability to adapt and respond to changing circumstances led to implementation difficulties.

**Conclusions**

For effective scaling up of PMR programmes, the provision of technical support and adequate resources are vital, but not sufficient on their own. An active strategy to manage relationships between implementing actors through effective communication mechanisms is essential. Successful outcomes may be realised if a strong and transparent management system, including management of financial resources, is put in place. This study provides evidence of the value of assessing implementation processes as part of impact evaluation for public health programmes.

## Background

Prompt treatment with effective anti-malarial therapy remains the cornerstone of malaria control in sub-Saharan Africa [1,2]. The home-management of malaria (HMM) strategy aims to improve prompt and effective anti-malarial drug use outside the formal health system [3], with a potential channel being the Private Medicine Retailers (PMRs) [4-6].

Interventions to promote early treatment of childhood fevers through PMRs have been tested in small scale settings [6] with a recent assessment in Kenya of the impact of PMR interventions at scale [7]. All these evaluations show that PMR interventions led to improved knowledge and practices of PMRs, and a high rate of appropriate treatment of malaria and other childhood illnesses among communities. However, past evaluations of PMR training programmes have focussed only on impacts on retailer knowledge and practices, providing limited evidence about how these impacts are influenced by implementation processes [6]. This paper, therefore, seeks to address this knowledge gap, drawing on the qualitative component of a larger study that assessed the performance of three different PMR programmes in Kenya, all of which involved efforts to scale up PMR training from small-scale pilot activities to district-wide implementation.

The three programmes were implemented by the Ministry of Health (MoH) in Kwale district; by a collaboration of government health officials and a non-governmental organization (NGO), Merlin, in Kisii Central district; and by a collaboration of government health officials and NGO, African Medical Research Foundation (AMREF), with support from the United States Agency for International Development (USAID) in Bungoma district. Full implementation began in 2005 and 2006 in Kwale and Kisii, respectively; but the Bungoma district programme has a history dating back to 1998, with a new phase of intervention activities implemented in 2005 after the withdrawal of the first line drug, Sulphadoxine pyrimethamine (SP). The current evaluation was conducted six months after the implementation of the programmes in each site.

The quantitative outcomes of these three programmes were assessed through a quasi-experimental design, involving comparisons between intervention and control areas, matched on the basis of similarity in geographical features, and malariometric indices. The primary indicator for the quantitative assessment was change in PMR knowledge and practice, following training on the use of amodiaquine as the drug of choice in the retail sector. As reported in detail elsewhere [8], positive effects were found in two sites. In Kisii, 60.5% of trained PMRs sold amodiaquine medicines in adequate doses compared to 2.8% in the untrained ones (OR; 53.5: 95% CI 6.7, 428.3). There was evidence of a lower impact in Kwale, where 18.8% of trained PMRs sold amodiaquine medicines adequately compared to 2.3% of control PMRs (OR; 9.4: 95% CI 1.1, 83.7). The study was unable to show evidence of impact in Bungoma as the scaling up process in this district prevented identification of suitable control areas. However, previous assessments had indicated broader impacts, where the intervention seems to have had a significant impact on stocking patterns, malaria knowledge and prescribing practices of shops/kiosks [9].

This paper compares implementation experiences across all three sites, considering what factors are likely to have contributed to differences in programme experiences, including the outcome differences between Kisii and Kwale districts.

## Methods

The study reported here was a retrospective policy analysis that investigated the three programmes’ implementation process. This involved data collection, both at district level and in the sub-district programme intervention areas. In Kwale, this included two out of the six divisions of the district; in Kisii, one out of eight divisions; and, in Bungoma, one out of ten divisions. Divisions are the third administrative tier in Kenya and have an average population of about 70,000 people.

Kisii Central is located in Western Kenya with a highland/epidemic type of malaria transmission. The district, which relies on agricultural activities, has a population of 491 786 people served by 140 health facilities. Bungoma is also situated in Western Kenya and predominantly agricultural, but with an endemic malaria profile. It has a population of 876 491, served by 61 health facilities. Kwale district is on the Kenyan Coast with an endemic malaria transmission pattern [10] and a population of 496 133, served by 109 health facilities. The district relies on tourism and fishing as the main economic activity.

### Data collection methods

To understand the policy and programme context, the Principal investigator (TA, first author) reviewed documents such as reports, work plans, minutes of meetings, memos, financial returns and programme materials. A document review template was used to collect relevant information on the content of the intervention, policy environment, actors involved and the implementation processes. These data were used to develop tools at the initial stages and to validate data from interview materials as part of triangulation. The process also helped to construct a time line of the implementation process.

Respondents for focus group discussions (FGDs) were selected purposively from the programme sites. For the client FGDs (n=12, four per district), the criterion for selection was mothers with children under five from the programme areas. For the retailer FGDs (n =12, four per district), the criteria were that the PMR was trained and was still operating a retail outlet within the programme area. All FGD respondents were recruited by local administrative leaders in each site, with the help of program officers. TA and two research assistants conducted all the FGDs. The number of participants varied in each group across the districts, averaging between 8-12 persons per session. All discussions were conducted in convenient venues for the participants. An attempt to capture group interaction was made through recording non-verbal expressions. All the interviews were recorded within the boundaries of confidentiality agreed at the time of discussions, and supplemented with written notes. All discussions were conducted in languages understandable to the participants, and focussed on normal client-PMR interactions, views on programme activities, goals, perceived barriers and facilitating factors.

In-depth interviews were conducted with 8 community volunteers, selected to support training and monitoring programme activities, and referred to as co-trainers (1 in Kisii, 4 in Kwale and 3 in Bungoma). Six main trainers (1 in Kisii, 2 in Kwale and 3 in Bungoma), public health officers-government officers with three year tertiary training on environmental health, were then interviewed. These interviews allowed an exploration of implementation experiences at the point of delivery. For clarification, issues raised from both sets of interviews were followed up during seven interviews with district level officers and programme managers. The focus of the manager interviews was on the role of actors, views on programme goals, implementation experiences and perceptions of factors influencing that process.

Finally, TA kept a field diary of daily activities in each district. Emerging issues and ideas were recorded, and detailed notes kept of activities and informal conversations with key actors. This enhanced reflexivity [11],supported transparency in the interpretations and formation of judgement, and enabled triangulation with other data during the analysis stage.

### Ethical considerations

Data collection was planned around local community timetables and took local events into consideration. The research aim and processes were explained to all participants, and their informed consent obtained for participation, recording of interviews, and use of verbatim quotes, where applicable. Ethical clearance was obtained from the National Ethical Review Board (Kenya Medical Research Institute (SSC no 1056).

### Data analysis

Qualitative interviews were taped, transcribed, and translated into English. Qualitative data were analysed using Nvivo7 (QSR international). Analysis entailed open coding and progressive categorisation of issues in an iterative process that included debriefing sessions with respondents and regular consultations with the research team [12]. The data were further organised along the policy analysis dimensions of context, content, actors and processes, proposed by Walt and Gilson [13]. Within each of these broader categories, more specific analyses were conducted using two additional frameworks: the scaling up framework, derived from consideration of experiences of public health innovations in low and middle income settings [14]; and a framework on factors influencing the diffusion of innovation, drawn from a systematic review of evidence from high income countries around the introduction of technology, products or other health care innovations [15].

These two frameworks identify five main areas of influence over the successful uptake of an innovation, as summarised in Table 1. The term “innovation” denotes new practices or set of interventions directed to improve quality of care through coordinated actions [14], and can be conceptualised as having two distinct but interrelated aspects. The first aspect consists of the central elements of the innovation, referred to as the “hard core”; and the second is the organisational structure and system, including management required for implementation, referred to as the “soft periphery” of the innovation [15]. The “resource team” are the individuals or organisations that have been involved in the development and testing of innovations and seek to promote its wider use. “User organisation” refers to the recipient of the intervention [14], which in this study is the district health system and district level public sector managers.

**Table 1:** Categories of issues likely to contribute to successful implementation

Categories	Relevant issues to consider
Attributes of innovation	Room to modify the innovation to suit local needs Ability of end users to see results of intervention

Attributes of the resource team	Effective leaders that command authority and respect Have understanding of the socio-economic environment of implementation. Training capacity Close physical proximity to the resource team

Attribute of the user organization	Have appropriate technical capacity to implement it. Stable and Effective leadership Perceives need for the innovation Linkages and networks both inside and outside the organization are more likely to ensure innovations become part of the user system

Scaling up strategy	Strategies that allow learning and managing local context

External Context	Taking advantage of policy windows such as drug policy changes with adequate communication Adapting to implementation environments (bureaucracy, socio- economic and cultural contexts).

Successful uptake of an innovation also depends on scaling up and organizational strategies. For example, if innovation implementation is led by a central government authority, like the MoH, it may be constrained by the rigid management processes of public bureaucracies that do not allow flexibility or take account of local context during implementation. An innovation is also less likely to be adopted if the resource team is dominated by experts and donors, in comparison to those where there is more participation by the user organisation [14]. Alternatively, uptake may be affected by the pace at which an innovation is implemented; and, gradual expansion through adapting the innovation in response to local realties is often important for success. Finally, responsiveness to changes in context is key to success. For example, political directives may include a “policy push” in the initial stages of implementation, boosting chances of success by making financial resources available for implementation [15]. Taking advantage of such policy windows may enhance uptake, although long term sustainability may be dependent on resources and sustaining the enthusiasm after the policy window is over [14].

## Results

Using the concepts outlined above, the experience of the three programmes around these two innovation aspects are outlined below.

### Hard core elements of the intervention

From the document review and interviews, five main programme activities were identified across sites: training, demand creation, accreditation, motivating actors, and monitoring and evaluation. For each programme, the details of these activities are outlined in Table 2, together with the implementing agencies and funders. Although similar programmes were implemented across sites, there were clear differences in specific activities, such as monitoring and evaluation (none was undertaken in Kwale, in comparison to the wider activities of the other two sites), as well as in training, and in demand creation.

**Table 2:** Key design features of programmes across sites

Characteristics	Kwale-MoH	Kisii-Merlin	Bungoma-AMREF
Training PMRs and their suppliers	Two-day participatory training workshops of PMRs to build their capacity	Three-day participatory training of PMRs to build their capacity	Social marketing, training of Mobile vendors, wholesale attendants, to disseminate job aids and build their capacity

Creating demand from consumers for appropriate drugs	Raising community awareness through supply of IEC materials to PMRs for distribution	Raising community awareness through public meetings, schools and churches and distribution of T-shirts with messages on fever management at community level	Raising community awareness through pyramid process, using drama, songs and community contacts

Accreditation of trained outlets	Provision of paper posters to enhance their credibility in the eyes of consumers	Provisions of wooden posters and award of certificates to enhance their credibility in the eyes of consumers	Enhancing their credibility through signing agreement forms, letters of approval and providing copies of the official government notice on recommended drugs

Motivation of actors	Motivating actors participating in workshops through financial token. Per diem allowances of $ 3.7 given to PMRs, and $ 5.2 for PHOs and DHMT members participating in the workshops.		Financial token and recognition through public meeting. $7.5 was given to mobile vendors and PHOs and DHMT members and recognition certificates to trainers and PMRs who performed well in the intervention during public meetings dubbed “Malaria Effective Treatment Nights”.

Monitoring and evaluation	None	Record keeping, quizzes and operational research supplemented with dialogue to solve problems encountered and on site reminders to promote sustainability of the new knowledge.	Through quizzes and operational research to sustain knowledge gained and assess areas for further improvement, receipts on distributed IEC materials collected to assess coverage.

Implementing agencies/funding	MoH –Global Fund	NGO-Merlin in collaboration with MoH with funding from Government of Finland	AMREF, USAID, QAP through contractual agreements between AMREF and CDC

In terms of training, in Kwale and Kisii, the intervention involved direct training of PMRs during participatory, skills-based workshops. The training covered information on malaria treatment and control; signs and symptoms of malaria, including danger signs; communication skills; and referral practices. In contrast, the Bungoma intervention did not have direct training of PMRs. Instead, it was primarily as a social marketing strategy, with one day’s training initially provided only for wholesale outlet and pharmacy owners to mobilise their support and enable them to engage with PMRs. The training provided wholesale outlet and pharmacy owners with information on the use of anti-malarial medicines and on the job aids that they were then intended to distribute to retailers in the periphery.

Based on early evaluation of its own passive dissemination strategy, the Bungoma programme developed a new information education and communication (IEC) aimed at demand creation activity, and called the *Jirani kwa Jirani* (JKJ- a Kiswahili phrase meaning neighbour to neighbour). This activity was based on a pyramid information dissemination approach in which trainers passed information and IEC materials to an initial group of community members, who then passed the same messages to five neighbours, who in turn trained five more neighbours – with the process repeated until all village members were covered. More active IEC dissemination strategies were also applied in Kisii district, compared to a more passive approach in Kwale.

Closer examination of innovation attributes through the lens of innovation theory (as summarised in Table 1) highlights, in particular, how in the Kisii programme, modifying the intervention to the local context supported positive outcomes. Unlike in Kwale, the face to face PMR training was planned around the community timetable to take account of socio-economic and cultural activities, such as farming and funerals, which affected the number of workshop attendees or their availability during follow up activities. These modifications resulted in timely supervision of PMRs, ensuring that the knowledge gained during initial training was sustained. In addition, and only in the Kisii programme, record keeping by PMRs allowed them personally to monitor trends in the cases they treated, and so enabled them to make informed opinions on when to build stock. As one retailer pointed out: *“It enables you to know a period when you need to stock more drugs from the record that you have kept”*.

### Soft periphery

#### Attributes of the resource team and user organization

In each site, the intervention was implemented by a combination of actors from outside and within the district health system (Table 3). Actors outside the district health team essentially provided technical support to the district health managers and are referred to here, using the language of innovation theory, as the resource team. The district health managers, meanwhile, are referred to as the user organisation, and were responsible for implementation. In addition, in all sites, individuals drawn from both groups, and referred to as a core team, acted as a bridge between the resource team and the user organisation, and sought, in particular, to harness support for the intervention from the user organisation.

**Table 3:** Program actors across the sites

Site	Resource team	User organisation	Core team
Kisii	Merlin project coordinator (overall supervision); Programme managers, IEC Officers, Monitoring and evaluation officers, field team	District Public Health Officer (coordination, logistical support, supervision and awareness creation); District Medical Officer (supervision); Divisional Public Health Officers (facilitators and trainer)	Three Merlin officials and three PHOs from the programme site

Kwale	Government officers from the Division of Malaria Control (DoMc), (strategic policy direction and facilitating funding); Researchers (technical support).	District Public Health Officer; District Medical Officer; Divisional Public Health Officers all with similar roles as described above	District PHO

Bungoma	USAID officials (designed and funded programme); Centres for Diseases Control (CDC) officials (part of initial team that designed the intervention and conducted operational research); AMREF officials (technical support); IEC specialists (designed and developed IEC materials).	District Public Health Officer; District Medical Officer; Divisional Public Health Officers with similar roles like Kisii	Five members from resource team and five different district health departments (nutrition, medical records, pharmacy, public health, social work)

Again, examination of programme experiences and, particularly, actors, through the lens of innovation theory (Table 1), highlights several influences over programme implementation and outcomes. First, the Kisii experience illustrates how the attributes of the resource team contributed to positive programme outcomes. In this site, the resource team had a long experience in supporting district activities, such as epidemic management of malaria. The success of these programmes generated a positive image of the resource team among the user organisation, enhancing Merlin’s credibility, a feature noted in innovation theory as necessary for success (Table 1). Members of the resource team had experience in strategic thinking and operational research around the role of PMRs in malaria control. As one member illustrated, ‘*I have been involved in the shopkeeper training programme for a long time and some of the challenges I have already gone through them in other settings. So it was quite easy for me to have a picture of where the programme is going, the challenges you are likely to encounter and how we have dealt with them in other settings”.* The resource team were also residents in the district, enabling frequent contacts with the user organisation and so building capacity through technical support, as well as allowing the development of a relationship of trust.

In contrast, the resource team in Kwale was a partnership between national stakeholders and researchers, neither of whom had a physical base in the district. In Bungoma, meanwhile, although the large resource team harnessed considerable technical support for the intervention, a feature that can facilitate innovation adoption (Table 1), only some members of the resource team, especially AMREF actors, were residents in the district. Moreover, the high mobility of the international members generated tensions and financial expectations, leading to interpersonal conflicts with the user organization that affected the implementation process and outcome.

Second, despite deliberate efforts to build user organisation capacity, a key challenge to implementation in Bungoma and Kwale was the transfer of key actors out of the districts, negatively affecting leadership stability (Table 1). As a member of the Bungoma user organisation noted: *“the challenges of implementation were because most of the District Health Management Team (DHMT) members who were there then were transferred, apart from one. The new people who came in had no idea about this, so adapting to that was a big issue; there was no continuity after the changes”*.

Third, only in the Kisii programme was there evidence that the programme responded to the felt needs of the user organisation, perhaps contributing to a supportive user environment (Table 1). As noted by a member of the user organisation: *“In Kisii Central, this programme took place in Kiamokama and Mosocho. These areas were facing the greatest malaria problems according to the International Centre for Insect physiology and Ecology (ICIPE) research, which was directing Merlin to areas where malaria was a big problem’*.

Fourth, all the programmes were linked to networks of actors. In innovations, such networks often provide resources that facilitate implementation and contribute to its sustainability [15]. In Kisii, formal networks at the district level between Merlin, the District Health Management Team and ICIPE, as well as intermittent temporary cooperation with other NGOs working in the district, enabled resource sharing and access to wider technical expertise. For example, ICIPE’s technical expertise in vector control research allowed Merlin to target the intervention geographically, as described above, on the basis of evidence. At the community level, moreover, Merlin linked with administrators, communities and retailers through sharing information and establishing committees to mobilize communities and strengthen grassroots support. Generally, these networks in Kisii resulted in effective use of programme resources and technical support, as well as strengthening PMRs and community support.

In contrast, few efforts were made in Kwale to develop partnerships with other organizations as the implementation strategy emphasised the user organisation’s role in managing the programme locally as a way of strengthening sustainability. In Bungoma, meanwhile, there was a much more complex network with many partners. At the national level, USAID developed linkages with AMREF through a formalized, contractual agreement. USAID also contracted the Quality Assurance Project (QAP) and Centre for Diseases Control (CDC) to provide technical expertise to the programme, leading to an intervention with many external partners working through the government system. However, the strong research role of CDC generated negative perceptions of the programme among the user organization because it was perceived to prioritise research over programme implementation. The presence of well resourced external partners in this instance contributed to destabilizing relationships at the district and community levels.

Finally, as Table 3 emphasises, the core teams were an important feature of all these programmes. In innovation theory terms, the core teams were intended to be the programme champions, facilitating implementation and strengthening linkages between the resource team and the user organisation [15]. In Kisii, the core team comprised experienced actors who were, as one noted, able to support implementation: *“You know different communities have different attitudes and my longer stay here has prepared me well to understand the type of people I deal with so that when I go there, I know the type of mechanism to put in place so that I can win them. So I think that enabled me to achieve a lot”*. In the other sites, however, the constitution and composition of this team generated tensions within the user organisation. The selection of only one divisional PHO as the core team in Kwale limited district wide support, whilst in Bungoma the lack of involvement of divisional actors caused problems. As a member of the user organisation noted: *“Actually because it is like they involved people at the DHMT level. But the PHO were brought on board later. And I think that is what they [implementing team] didn’t take nicely..... We didn’t do it wisely, the composition varied ...It is unfortunate that it is just the DHMT that took over, and we didn’t realize that at the end of it we will need other people who are the real stakeholders”*.

### Managing the implementation of the innovation

Whilst innovation theory highlights the influence of a range of strategy issues on implementation success (Table 1), programme experiences highlight, instead, important management factors. These sets of issues are considered together here in four categories: the scaling up strategy, actor management, adapting to context, and financial management.

#### The type of scaling up strategy

In Kisii and Kwale, the implementation process began in selected geographical divisions, with the aim of covering the entire district through the gradual expansion of activities to other areas, illustrating a horizontal scaling up approach [14]. This process enabled the careful implementation of all elements of the intervention. The gradual expansion of the intervention also allowed personal contact to develop with PMRs, enhancing the communication and feedback process, and identifying gaps in retailers’ knowledge which could be addressed. However, as the main responsibility for implementation fell on the user organisation in Kwale, other competing district level activities sometimes disrupted the implementation process. For example, the national measles campaign in 2005 interrupted divisional level actors mobilisation of the programme, and resulted in limited demand creation at community level.

In Bungoma district, meanwhile, implementation occurred on a district-wide basis, and scaling up sought to expand programme coverage by adding a new element (community awareness through the JKJ strategy) to the existing intervention. This is referred to as a functional diversification or grafting scaling up strategy [11]. This approach, however, faced the challenge of how to sustain the new activities when external funding came to an end. In addition, the withdrawal of funding without consultation resulted in a disruption of the activities among the user team and communities, undermining their support of the intervention.

#### Managing actors and wider context

The implementation experiences demonstrate that relationships between the actors involved in every programme required deliberate management, and there was clear evidence that failure to manage these relationships affected implementation processes. For example, in Kwale, unrelated tensions over financial accounting among the user organisation members led to delays in convening DHMT meetings. This delayed implementation as one condition of the funding was to convene a DHMT meeting before any money was spent. On the other hand, a good illustration of a deliberate actor management strategy was noted in Kisii. Relationships between the resource team and user organisation were managed by a memorandum of understanding (MoU), introduced in the initial stages of implementation by the resource team. The MoU spelt out the roles of the resource team and the user organisation, and how resources would be shared. This MoU enabled constructive participation in intervention planning by both groups, resulting in successful implementation of most intervention elements. These characteristics were absent in the other sites.

In Kisii, programme implementation was driven by action research, and this allowed the intervention to be adapted to the local environment. Decision making was also decentralized to the local Merlin offices, enabling local initiative, mutual learning and problem solving. Although action research was also used to guide implementation in Bungoma, adaption to local context was minimal, largely due to coordination challenges between the user organization members in relation to how the data was used for planning. In Kwale, meanwhile, implementation was managed by the user organisation through the much less flexible decision making processes of the government bureaucracy.

The strength of management in the Kisii site was demonstrated by the deliberate efforts made to contain the impact on programme implementation of the national level decision to move from sulphadoxine pyrimithamine (SP) to artemisinin based combination as the first line drug for malaria treatment in 2004. Initial information on drug policy changes was relayed through formal government procedures; however, given a lack of clarity on the drug of choice for the retail sector, Merlin actively sought, through its own networks, further information and official documentation on this issue. The MoH then sent out a circular stating that PMRs could be trained on amodiaquine as the over the counter medicine until a proper policy was developed, allowing the resource team to revise all its IEC materials before the policy change occurred.

In the other two sites, however, the drug policy change had negative influences on programme implementation. In Kwale, inadequate communication through official government channels led the DHMT to perceive that central government did not actively support the involvement of PMRs in malaria control. The slow response in communicating on the type of medicine to be used for the retail sector also created concerns over the continuity of the intervention among the user team. This generated challenges such as inadequate distribution of materials that had already been produced with SP medicines. Revision and production of new IEC materials referring to the new drug of choice could then not be undertaken because of budgetary constraints. In Bungoma, meanwhile, although AMREF, like Merlin, had national networks, it did little to secure clarification on the drug of choice for the retail sector in time to adapt its activities. Furthermore, as the JKJ strategy had emphasised SP, the drug policy change eroded the confidence of trainers and challenged the authenticity of information passed to clients, affecting delivery of the intervention.

#### Financial management and its influence on process and outcome

The process of financial disbursement mirrored the organisational arrangements of the organisations supporting each programme. In Kisii, the resource team was not only well funded but worked within an efficient system of disbursing funds for implementation. The flexible funding process facilitated changes in some hard core elements of the innovation. For example, in response to suggestions from PMRs on the need for more time to understand the content of training, the resource team prolonged the training period from two to three days. This promoted understanding and contributed to improved knowledge and PMRs practices compared to the other sites.

In contrast, Kwale had a complex funding process, with lengthy timelines, that was tied to the government financial management system and constrained implementation, a feature that was particular to Kwale and not necessarily associated with other MoH programmes. Moreover, the failure to harmonise divisional level budgets with the district work plans meant that the budgetary needs were not fully met, or prevented funds from being used for local adaptation. This led, in particular, to inadequate demand creation, as illustrated here: *“Mobilization was not extensively done because according to the plan, there was only a day or so for mobilizing the communities. Now you understand that some of areas are extensive that you cannot cover in a day or so many of the areas were not extensively sensitised of the programme.”*

Finally, in Bungoma, all funds were disbursed to AMREF through a contractual agreement with USAID. The resource team in Bungoma therefore had direct control of programme funds, and this facilitated timely implementation of activities, as well as allowing programmatic adaptations to local realities. However, as the DHMT had no role in financial management this, combined with financial expectations among programme implementers, generated the perception of a lack of transparency and wider tensions: *“One of the biggest challenges was financial resources because people were not transparent. You see if we are partners and if you want us to have active participation then it is also good that people know what the inputs are. People did not know what went into this program for the five years if they knew then they read about it in the final reports. I think people were in the dark. This made people suspicious, and the credibility of the good project was damaged so people always feel they were being cheated’* (member, user organisation).

## Discussion

This study has firstly demonstrated the range of factors that influence the scaling up of PMR programmes and their successful implementation and, then, highlighted key approaches that can support successful scaling up of such programmes. The main limitation is that the study was not set up to test propositions about the factors explaining implementation problems and achievements derived from these frameworks. Nonetheless, the use of the frameworks in analysis helped guide systematic consideration, both within and across sites, of the factors that wider theory and empirical evidence suggest can facilitate or hamper effective implementation of innovative programmes. This process enabled reflection on explanations for the observed variations across programmes in implementation experience, including the primary outcome variable.

The factors shown to influence PMR implementation are confirmed by wider experience. Many studies highlight the important influence of actors over the successful implementation of health programmes. For example, in Kenya, the introduction of the Malarone® donation programme showed the political and bargaining influence of national, regional or international actors over the development and implementation of the programme. International actors did not effectively involve national bodies responsible for drug policies, and there were many differences of opinion between regional bodies and international actors over the relevance of donation as a policy intervention. As a result, the programme was characterised by conflicts in its early stages and was only slowly implemented [16].

The influence of implementing actors, specifically, on policy implementation, is well documented in studies from various settings. Front line providers have been shown, for example, to understand and implement policies differently from original intentions, often undermining effective implementation [17-19]. In addition, experiences from other settings show how instability in local health system leadership negatively influences policy implementation. In Brazil, for example, leadership changes following elections stalled the implementation of family planning services and threatened the sustainability of reproductive services [20]. Similarly, in Zambia, health care reforms led providers to feel insecure in their jobs, resulting in a high turnover of staff during the implementation of new reproductive services and undermining the scaling up of contraceptive services [21].

Wider evidence also highlights both the challenges of managing through inflexible government bureaucracies, and the importance of flexible management systems in successful scaling up processes. Often, the centralised bureaucratic procedures of government systems undermine local decision-making flexibility and so present challenges to the implementation of innovative interventions [14]. For example, in a study aimed at illuminating the difficulties of implementing reproductive health programmes in Kenya, Ghana, Zambia and South Africa, the authors illustrate how financial decision making remained subject to centralised controls, even in the context of a decentralised system in Ghana and Zambia. This made the integrated management of resources responding to local needs and priorities difficult [22].

**Figure 1:**
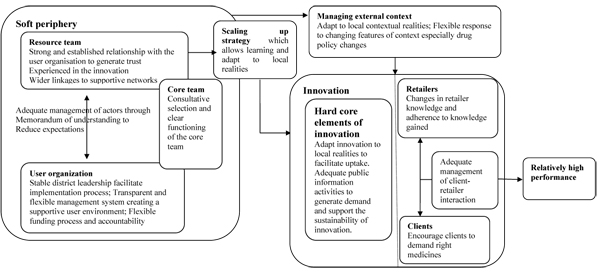
summary of key factors enabling successful implementation of PMR interventions

Drawing on the study findings, Figure 1 summarises the set of factors likely to facilitate the successful scaling up of PMR training programmes. It shows that successfully introducing PMR interventions requires that attention be paid not just to the overarching programme design but also to the features of the resource team, user organisation and their relationships, which are also important influences over effective programme implementation. These elements of the soft periphery of an innovation [14] have been largely overlooked in planning PMR interventions.

Given the particular importance of actor management and flexible implementation processes, three main conclusions can be derived from this study for future implementation of PMR training programmes. First, it is important to appoint skilful leaders to drive scaling up processes. A strong and experienced resource team that has a good relationship with the user organisation is likely to be trusted within the local setting, and supports effective scaling-up. In addition, where this team has links into wider networks, additional informational and other resources can be drawn into supporting programme implementation. This conclusion has implications for the types of people that might be important in resource teams, and for the time that must be invested in setting up the relationships supporting policy implementation. Continuity in the implementation of PMR programmes is also dependent on the stability of district leadership. Moreover, in all three districts examined here, a core team (Figure 1) was established to lead implementation. Establishing such a team is widely recommended in the PMR strategic documents [23]. This experience shows that the core team worked better when a consultative process, involving the user organisation and the resource team, was used in selecting its members, and when these members had a good understanding of the management systems of both the resource team and the district health system. Where core team members are drawn from a range of organisations, it is also particularly important to clarify carefully their roles and responsibilities to avoid confusion and duplication of activities.

Second, the study illustrates the importance of building and managing partnerships as part of intervention management, as recommended by World Health Organisation (WHO) guidelines for HMM programmes [24]. Experience from previous PMR interventions supports the recommendation of involving public health officials and community representatives in curriculum development, training and supervision, as this is likely to contribute to acceptability [6]. Recent documentation on PMR programmes also suggests that to enhance the chances of programme acceptability, the community entry process should involve key figures from social, political, and religious groups, and should sustain the interactive nature of consultation and negotiation with stakeholders [23]. While this study’s findings provide evidence of the value of involving a wide spectrum of actors in planning and development, it also highlights the challenges that result from the involvement of many actors. Careful management of these actors, for example, through effective communication mechanisms, and particularly of the relationship between the resource team and user organisation, is likely to improve relationships between these actors. Use of an MoU that provides a framework for offering incentives to all actors involved, and clarifies the roles of different partners in the implementation, as seen in Kisii, is likely to be important.

Finally, the study provides ideas of how to address the challenges imposed by various features of context. To avoid the implementation gaps experienced in Kwale, future implementation of PMR programmes may need to find ways to allow greater authority in budgetary decisions at a local level, even within existing government procedures. Such authority brings the flexibility necessary to allow the user organisation to experiment with adapting different elements of PMR to local contexts, supporting positive outcomes (Figure 1). The presence of a resource team working to support PMR implementation from outside government structures but in close collaboration with the user organisation, as in Kisii, may provide such flexibility in some settings. However, this is less possible when funds for programme implementation are channelled through government systems to actors such as district health systems. In such situations, it is important that there is sufficient decentralisation of decision-making authority to the local level, supporting flexible management action that is responsive to local needs and circumstances. Such flexibility may also allow responsiveness to broader policy changes which influence the programme, such as changes in drug regimens. However, it is equally, if not more, important that the implementation of any policy change likely to impact on PMR programmes is thought through carefully at a national level so as to limit negative impacts on local activities. In this study, the Kisii case study illustrates positive outcomes with fewer challenges of implementation than in the other cases. This seems likely to be associated with the particular management approach adopted in this site, and its flexibility.

## Conclusions

Overall, the study shows that for effective scaling up of PMR programmes, the provision of technical support and adequate resources for successful adoption of the PMR intervention by the user organization are vital, but not sufficient on their own. Deliberate and careful management of the implementation process is key to successful uptake and impact at retailer level. Three keys aspects of management are critical for success. First, it is important to develop an active strategy to manage relationships between implementing actors through effective communication mechanisms, and by early, clear and transparent development of a set of principles to guide relationships (as the MoU in the Kisii cases study illustrated). Second, key actors responsible for implementation must have adequate management training and support. Third, successful outcomes may be realised if a strong and transparent management system, including management of financial resources, is put in place. This study, finally, provides evidence of the value of assessing implementation processes as part of impact evaluation for public health programmes, and the role of policy analysis approaches. Such assessment enhances the interpretation of quantitative outcomes and provides a basis for generating lessons about how to strengthen future implementation.

## Competing interests

The authors declare that they have no competing interests.

## Authors' contributions

TA was involved in the conceptual design of the study, collected and analysed the data and was responsible for the overall writing of the manuscript. AA was involved in the study design and revising the manuscript for intellectual content. WA provided information on policy discussions around the retail sector strategy and revised the manuscript. VM conceptualised the study and was involved in the overall design and writing the manuscript. LG was substantially involved in the study design and revising the manuscript for intellectual content.
